# Hydroxyl-substituted double Schiff-base condensed 4-piperidone/cyclohexanones as potential anticancer agents with biological evaluation

**DOI:** 10.1080/14756366.2018.1501042

**Published:** 2019-01-02

**Authors:** Lianshuang Zhang, Qin Chen, Guige Hou, Wei Zhao, Yun Hou

**Affiliations:** a School of Basic Medical Sciences, Binzhou Medical University, Yantai, P. R. China;; b Department of Pharmacy, Taihe County People’s Hospital, Taihe, P. R. China

**Keywords:** 3,5-*Bis*(arylidene)-4-piperidone, anticancer activity, cyclohexanone, Schiff-base

## Abstract

Novel hydroxyl-substituted double Schiff-base 4-piperidone/cyclohexanone derivatives, **3a–e, 4a–e, 5a–d,** and **6a–c**, were synthesized and fully characterized by ^1^H NMR, IR and elemental analysis. The cytotoxicity against human carcinoma cell lines A549, SGC7901, HePG2, HeLa, K562, THP-1 and non-malignant LO2 cell lines were evaluated. The results showed 4-piperidinone derivatives displayed better cytotoxicity than cyclohexanone derivatives, especially for 3,4,5-trihydroxyphenyl-substituted BAP **5c**. The western blot and flow cytometry results proved **5c** can effectively promote cell apoptosis through up-regulating Bax protein and down-regulating Bcl-2 protein expression. Molecular docking modes showed that **5c** could reasonably bind to the active site of Bcl-2 protein through strong intermolecular hydrogen bonds and significant hydrophobic effect. *In vivo*, **5c** can effectively suppress the growth of HepG2 xenografts without apparent body weight changes. This study indicates that **5c** can be a potential anticancer agent for early treatment of liver cancers.

## Introduction

**Figure 1.**
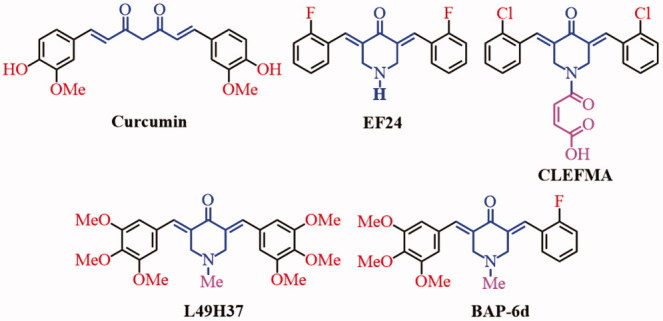
The structures of curcumin and some BAPs.

Curcumin (Figure 1), a major active component of the food flavor turmeric, has anti-inflammatory, antibacterial, anticancer and antioxidant activities^1^. Curcumin and its analogues containing the pharmacophore of 1,5-diaryl-3-oxo-1,4-pentadienyl, are thought to interact at the primary binding site, bio-thiols from susceptible neoplasms. Another pharmacophore of methoxyphenol groups at an auxiliary site also can influence their bio-activities. However, due to its low aqueous solubility, instability and low bioavailability, the clinical utility of curcumin is limited^2^^,^^3^. In recent years, researches have been focused on improving the bioavailability through the structure modification of curcumin^4–6^. Some 2,6-dibenzylidenecyclohexanone and 3,5-*bis*(arylidene)-4-piperidone derivatives (BAPs) were synthesized and evaluated bioactivity. These compounds generally possess the 1,5-diaryl-3-oxo-1,4-pentadienyl pharmacophore into their structures to form one or more *α,β*-unsaturated keto groups, which can react preferentially or exclusively with thiols in contrast to amino and hydroxy groups resulting in a greater chemosensitivity to tumors rather than with normal cells^7^^,^^8^. In addition, two of *α,β*-unsaturated keto groups enable sequential attacks of cellular thiols to display the better activities^9^. 3,5-*Bis*(2-flurobenzylidene)piperidin-4-one (EF24, Figure 1) is a novel curcumin analog, which can inhibit tumor growth and metastasis by inhibiting NF-κB pathways^10^^,^^11^. CLEFMA (Figure 1) can inhibit growth of lung cancer xenografts through inhibiting anti-apoptotic markers (cellular inhibitor of apoptosis protein-1 (cIAP1), Bcl-xL, Bcl-2, and survivin) expression, up-regulating the pro-apoptotic markers Bax and BID expression, and inducing apoptosis by cleavage of caspases 3/9 and PARP^12^. Curcumin analog (3*E,*5*E*)-3,5-*bis*(3,4,5-trimethoxybenzylidene)-1-methylpiperidin-4-one (L49H37, Figure 1) with fertile electron-donating substitutes exhibits more potent inhibitory effects than curcumin against pancreatic stellate cells (PSCs) cell cycle^13^. In addition, nitrogen-containing heterocyclic dienones, such as 4-piperidone, can display higher inhibitory properties toward human carcinoma cell lines compared with their homocyclic dienone analogs (such as cyclohexanone)^14^.

Recently, some symmetric or dissymmetric *N*-substituted-3,5-*bis*(arylidene)-4-piperidone derivatives as anticancer agents were reported by our group^15,16^. In addition, some bispyridyl-substituted BAPs and their pharmaceutical co-crystals with the interesting luminescent properties^17^ and the improved anticancer activities were reported. The results showed that the improvement of anticancer activities should be related to the synergistic effect between BAPs and gallic acid^18^. Some dissymmetric BAPs with different substituent groups on both sides of BAP demonstrated potential inhibitory activities against HepG2 and THP-1, especially for the most active (3*E,*5*E*)-3-(2-fluorobenzylidene)-5-(3,4,5-trimethoxybenzylidene)-1-methylpiperidin-4-one (BAP-**6d**, Figure 1) induced HepG2 cells apoptosis in a dose-dependent manner by flow cytometry *in vitro* and suppressed the growth of HepG2 xenografts in nude mice and relatively non-toxic to mice^19^. *N*-phenylsulfonyl-BAPs can prevent the nuclear translocation of NF-κB induced by Lipopolysaccharide (LPS) or TNF-α and exhibit both anti-inflammatory and anticancer activities in hepatic carcinoma cell lines^20^.

In addition, Schiff-base compounds with active –C = N group can be a good donor site or an active ligand, which exhibits a range of biological activities, including antifungal, antiviral, anticancer, antibacterial and anti-inflammatory properties^21–26^. Our interests lie in incorporation of amino-substituted 3,5-*bis*(arylidene)-4-piperidone derivatives and phenolic hydroxyl-substituted aromatic aldehydes to construct phenolic hydroxyl-substituted double Schiff-base 4-piperidone/cyclohexanone derivatives with desired antitumor activity.

## Experimental

### Materials and methods

Cyclohexanone, *N*-methyl-4-piperidinone, 4-piperidone hydrate hydrochloride, tetrabutyl ammonium bromide (TBAB), stannous chloride and all aromatic aldehydes were purchased from Sinopharm Chemical Reagent Co. Ltd (Shanghai, China) and were used as obtained without further purification. Compounds (2*E*,6*E*)-2,6-*bis*(3-aminobenzylidene)cyclohexanone (**2a**) and (3*E*,5*E*)-3,5-*bis*(3-aminobenzylidene)-1-methylpiperidin-4-one (**2b**) were prepared based on a literature^25^^,^^26^. ^1^H NMR data were collected using a Bruker Avance 400 MHz. Chemical shifts were reported in *δ* relative to TMS. 13C NMR data were collected at 100 MHz on a Bruker Avance 400 MHz spectrometer, or at 150 MHz on a Bruker Avance 600 MHz spectrometer. Elemental analyses were performed on a Perkin–Elmer Model 240c analyzer. Infrared (IR) spectra were obtained in the 400–4000 cm^−1^ range using a Perkin-Elmer Frontier Mid-IR FTIR Spectrometer.

### Preparation of 2c and 2d

4-Piperidone hydrate hydrochloride (1.53 g, 0.01 mol) or *N*-methyl-4-piperidinone (1.13 g, 0.01 mol), and *m*-nitrobenzaldehyde (3.32 g, 0.022 mol) mixed with 40 mL glacial acetic acid. Dry hydrogen chloride gas was bubbled into this mixture for about 45 minutes, and the mixture continued stirring at room temperature for 24 hours (monitored by TLC). The precipitate was collected and dried under the vacuum to obtain crude intermediates 1c or 1d, which were untreated and directly used in the following reaction. And then, the crude intermediates 1c or 1d dissolved in a solution of water (20 mL) and methanol (20 mL) with TBAB (0.32 g, 0.001 mol). Stannous chloride (6.75 g, 0.03 mol) dissolved in concentrated hydrochloric acid (15 mL), was slowly added to the reaction system. The mixture was stirred at 40 °C for 8 hours. After cooling to the desired temperature, the precipitate was collected and washed with 5% sodium hydroxide solution, and dried under the vacuum to get yellow powder **2c** or **2d**.

### Preparation of 3a–e, 4a–e, 5a–d, and 6a–c

Compounds **2a** (0.30 g, 0.001 mol), **2b** (0.32 g, 0.001 mol), **2c** (0.34 g, 0.001 mol), or **2d** (0.35 g, 0.001 mol), and aromatic aldehyde (0.002 mol) were dissolved in methanol (5 mL). A drop of formic acid was added into the mixture. And the mixture was stirred for 3–8 hours at room temperature (monitored by TLC). The precipitate was collected and recrystallised by methanol to afford **3a**–**e**, **4a–e**, **5a–d**, and **6a–c**.

### Anticancer testing with MTT method

Compounds **2a–d, 3a–e, 4a–e, 5a–d**, and **6a–c** were screened against human neoplastic cell lines such as human lung carcinoma cells (A549), human gastric adenocarcinoma cells (SGC7901), human cervical carcinoma cells (HeLa), human liver hepatocellular carcinoma cell line (Hepg2), human chronic myelogenous leukemia cell line (K562), human acute mononuclear granulocyte leukemia (THP-1), human normal heptical cell line (LO2) (Table 1) using modified MTT assay (3-(4,5-dimethyl-2-thiazolyl)-2,5-diphenyl-2-H-tetrazolium bromide, MTT, Dojindo Laboratories, Tokyo, Japan). All cell lines were maintained at 37 °C in a humidified 5% CO_2_ and 95% air atmosphere. The HePG2 cell lines were cultured in DMEM containing 10% fetal bovine serum. Other cell lines were grown in medium 1640 and supplemented with 10% fetal bovine serum. The tested compounds and positive controls (Doxorubicin and Curcumin) were initially dissolved in dimethylsulfoxide (DMSO), and then the solutions were diluted 1:1000 in DMEM medium or 1640 medium, which final concentration of DMSO was always 0.1% (v/v). Controls were prepared with 0.1% DMSO (v/v) only. The cells were seeded in a 96-well plate in 200 µL medium per well at a density of 1 × 10^4^ cells/well for 24 hours. The cells were treated with serial concentrations of compound and incubated for 24 hours. Cells with only culture media were used as control. After the media was removed, 20 µL of MTT (5 mg/mL) was added then the plates were incubated for 4 hours at 37 °C in cell culture incubator. The MTT containing media were removed and then 150 µL of DMSO was added to dissolve the dark-blue formazan crystals. The optical density (OD) was measured by a multi-well plate reader (TECAN, Männedorf, Switzerland) at 570 nm. The results are expressed as a decrease in the cell viability (%) in comparison to untreated controls. The concentration of each compound was examined in triplicate, and the IC_50_ values are expressed in Table 1. The concentrations of the compounds were 50, 20, 10, 5, 2.5, 1, 0.5, 0.1, 0.05 and 0.01 µg/mL. Doxorubicin (DOX) and Curcumin were used as positive controls. The concentrations of DOX were 50, 20, 10, 5, 1.5, 1.0, 0.5, 0.1, 0.05 and 0.01 µg/mL. The concentrations of curcumin used were 100, 50, 25, 12.5, 6.25, 3.125, 1, 0.5 and 0.1 µg/mL.

**Table 1.** Cytotoxicity of BAPs, Curcumin and DOX.

Compd	A549		SGC7901		HePG2		HeLa		K562		THP-1		Ave		LO2
	IC_50_ (μM)	SI^a^	IC_50_ (μM)	SI^a^	IC_50_ (μM)	SI^a^	IC_50_ (μM)	SI^a^	IC_50_ (μM)	SI^a^	IC_50_ (μM)	SI^a^	IC_50_ (μM)	SI^a^	IC_50_ (μM)
**2a**	7.9 ± 0.1	3.6	9.4 ± 0.2	2.3	10.7 ± 0.5	2.6	25.9 ± 3.7	1.1	22.2 ± 1.9	1.3	6.2 ± 1.1	4.5	13.7	2.7	28.2 ± 3.9
**2b**	6.5 ± 0.1	2.6	4.2 ± 0.3	4.0	3.1 ± 0.2	5.4	4.4 ± 0.1	3.8	4.3 ± 0.1	3.9	1.5 ± 0.1	11.5	4.0	5.2	16.9 ± 0.2
**2c**	3.4 ± 1.2	5.5	2.5 ± 0.8	7.4	5.8 ± 0.2	3.2	2.2 ± 0.1	8.7	2.0 ± 0.1	9.3	1.4 ± 0.2	13.9	2.9	8.0	18.8 ± 1.4
**2d**	4.1 ± 1.0	5.4	3.8 ± 1.2	5.6	6.2 ± 0.3	3.5	3.1 ± 0.1	6.9	3.0 ± 0.1	7.3	2.1 ± 1.1	10.3	3.7	6.5	21.6 ± 2.8
**3a**	7.4 ± 0.3	2.5	11.4 ± 0.2	1.6	3.3 ± 0.1	5.6	9.9 ± 0.3	1.8	3.4 ± 0.01	5.4	1.5 ± 0.1	12.4	6.1	4.9	18.2 ± 0.2
**3b**	6.1 ± 0.4	2.8	2.7 ± 0.6	6.4	2.8 ± 0.1	6.1	8.9 ± 0.2	1.9	3.4 ± 0.01	5.0	1.6 ± 0.1	10.5	4.3	5.5	17.1 ± 0.1
**3c**	10.6 ± 1.1	1.1	5.2 ± 0.8	2.3	13.7 ± 1.1	0.9	11.4 ± 0.9	1.0	14.0 ± 0.1	0.9	5.3 ± 0.01	2.3	10.0	1.4	12.0 ± 0.1
**3d**	4.1 ± 0.6	2.8	7.1 ± 1.0	1.6	4.0 ± 0.1	2.9	13.1 ± 1.0	0.9	3.2 ± 0.02	3.6	3.2 ± 0.03	3.6	5.8	2.5	11.3 ± 0.1
**3e**	2.5 ± 0.4	4.4	3.0 ± 0.8	3.8	1.9 ± 0.01	6.0	12.8 ± 0.9	0.9	4.2 ± 0.1	2.7	3.3 ± 0.1	3.4	4.6	3.5	11.3 ± 0.3
**4a**	5.2 ± 0.4	2.1	8.3 ± 0.8	1.3	2.59 ± 0.10	4.1	6.3 ± 0.2	1.7	2.8 ± 0.1	3.8	0.9 ± 0.1	12.0	4.3	4.2	10.6 ± 0.1
**4b**	4.1 ± 0.4	3.4	1.4 ± 0.2	10.0	1.07 ± 0.1	12.9	3.5 ± 0.1	4.0	2.15 ± 01	6.4	1.2 ± 0.03	11.5	2.2	8.0	13.8 ± 0.1
**4c**	9.0 ± 0.8	1.4	3.0 ± 0.3	4.3	3.54 ± 0.1	3.6	4.7 ± 0.1	2.8	6.2 ± 0.7	2.1	2.5 ± 0.2	5.1	4.8	3.2	12.8 ± 0.2
**4d**	2.6 ± 0.5	4.4	2.3 ± 0.1	5.1	1.11 ± 0.1	10.6	4.1 ± 0.1	2.8	2.0 ± 0.02	6.0	1.0 ± 0.03	12.3	2.2	6.9	11.7 ± 0.1
**4e**	1.5 ± 0.3	7.2	2.0 ± 0.3	5.4	1.27 ± 0.02	8.6	2.6 ± 0.1	4.2	1.8 ± 0.01	5.9	0.9 ± 0.1	11.8	1.7	7.2	10.9 ± 0.2
**5a**	2.8 ± 0.2	6.4	1.6 ± 0.1	11.2	1.8 ± 0.05	9.7	1.1 ± 0.4	15.0	1.5 ± 0.1	11.7	1.0 ± 0.3	18.5	1.6	12.1	17.6 ± 1.0
**5b**	3.0 ± 0.1	4.8	1.3 ± 0.2	10.9	3.3 ± 0.2	4.3	4.2 ± 0.5	3.4	5.7 ± 0.4	2.5	2.2 ± 0.1	6.4	3.3	5.4	14.1 ± 1.0
**5c**	1.4 ± 0.2	10.7	1.4 ± 0.1	11.1	0.6 ± 0.1	24.5	1.5 ± 0.3	9.8	1.3 ± 0.1	11.8	0.9 ± 0.2	17.0	1.2	14.1	15.2 ± 0.6
**5d**	2.5 ± 0.1	5.8	1.8 ± 0.1	8.3	2.7 ± 0.2	5.6	3.8 ± 0.2	3.8	1.5 ± 0.1	9.7	2.1 ± 0.2	6.8	2.4	6.7	14.57 ± 1.26
**6a**	1.9 ± 0.1	11.6	2.4 ± 0.1	9.2	2.6 ± 0.1	8.4	2.2 ± 0.2	9.9	2.8 ± 0.2	7.7	1.5 ± 0.2	14.2	2.2	10.2	21.7 ± 2.0
**6b**	2.3 ± 0.1	9.1	2.6 ± 0.1	8.3	2.7 ± 0.2	7.8	3.4 ± 0.4	6.2	5.1 ± 0.3	4.2	1.8 ± 0.1	12.0	3.0	7.9	21.3 ± 2.0
**6c**	1.5 ± 0.2	12.5	2.0 ± 0.2	9.3	2.6 ± 0.2	7.1	2.5 ± 0.1	7.5	1.9 ± 0.2	9.5	1.6 ± 0.1	11.4	2.0	9.5	18.4 ± 1.0
Curcumin	19.4 ± 0.2	1.4	21.5 ± 0.3	1.3	16.0 ± 0.2	1.7	23.8 ± 1.3	1.2	22.1 ± 0.8	1.2	21.7 ± 0.4	1.3	20.8	1.4	27.5 ± 0.4
DOX	3.8 ± 0.1	1.6	7.0 ± 0.4	0.9	0.6 ± 0.1	10.4	2.1 ± 0.3	2.9	1.6 ± 0.2	3.9	0.5 ± 0.2	12.8	2.6	5.4	6.1 ± 0.5

^a^The letters SI refer to the selectivity index which is the quotient of the IC_50_ values towards nonmalignant LO2 cells.

### Western Blot assay

The HepG2 cell lines were treated with different dose of by BAP **5c** (1.0, 2.0, 4.0 µM, respectively) for 3 hours, then washed twice with PBS, and lysed in ice-cold modified RIPA buffer containing PMSF (RIPA:PMSF = 100:1; the ultimate density of PMSF is 1.0 mM). The lysates were kept for 30 minutes on the ice, and then centrifuged for 15 min at 12000 rpm/min at 4 °C and the supernatant was collected. The proteins were boiled at 95 °C at 5 min with loading buffer (the volume ratio of protein and the loading buffer is 4:1). Each 50 µg protein of cell lysates were separated by 10% SDS-PAGE gel electrophoresis. The proteins were then transferred onto polyvinylidene fluorides membrane. The membranes were probed with antibodies and visualised using an enhanced chemiluminescence (ECL) detection kit according to the manufacturer instruction.

### Molecular docking study

The protein structures were cleaned and inspected for errors and missing residues. Hydrogens were added, and the water molecules were deleted. The structures were assigned with Gasteiger-Hückel charges. Other parameters that are not mentioned were set at default values. The molecular docking of BAP **5c**, against Bcl-2 protein was performed using Discovery Studio 2017R2 (DS, Accelrys Inc., San Diego, CA, USA).

### Apoptosis assay

HepG2 cells were plated at a density of 1 × 10^5^ cells/well in 24-well plates. After treated with BAP **5c** (2 and 4 µM) and DMSO for 24 hours, the cells were harvested, washed twice with pre-chilled PBS and suspended in 1X binding buffer at a concentration of 1 × 10^6^ cells/mL. One hundred microliters of such solution (1 × 10^5^ cells) was mixed with 5 µL of Annexin V-FITC and 5µL of Propidium Iodide (BD Biosciences, San Jose, CA, USA) according to the manufacturer’s instruction. The mixed solution was gently vortexed and incubated in the dark at room temperature for 15 minutes. Four hundred microliters of 1X binding buffer were then added to each tube and cell apoptosis analysis was performed by flow cytometry (BD FACS Calibur) within 1 hour.

### Cellular uptake assay in HepG2 cells using CLSM

HepG2 cells were seeded in 6-well plates at a density of 2 × 10^5^ cells/well in 2.0 mL of culture medium and cultivated for 24 hours, and then incubated with **5c** for 1 hour, 2 hours, 4 hours, 8 hours, respectively. The cells were washed with PBS and fixed with 4% (w/v) paraformaldehyde for 1 hour at room temperature and 100 mL (10 mg/mL) Hoechst 33342 was applied to stain the nuclei for 15 minutes. Finally, the glass cover slips containing cells were mounted onto slides and observed under confocal laser scanning microscopy (CLSM, TCS SPE, Leica, Germany).

### *In vivo* antitumor efficacy

The antitumor effect was investigated on the seven-week-old female Balb/c nude mice (*n* = 30) weighing 19–21 grams (Shanghai Laboratory Animal Center). Briefly, 0.2 mL HepG2 cells (1 × 10^7^/mL) were inoculated subcutaneously into the alar left of each mouse. On the third day after the inoculation, the HepG2-bearing mice were randomly divided into five groups (*n* = 6/group) and administrated the according drug or solvent. For the treatment groups, BAP **5c** was dissolved in saline containing 1% DMSO, and doxorubicin (DOX) was dissolved in saline. BAP **5c** was daily administered by i.p. injection of (10 mg/kg/d, 1.0 mg/kg/d and 0.1 mg/kg/d) for 20 days, DOX (1.0 mg/kg/2d) was administered by every two days 1mg/kg for 20 days. The control group was administrated of saline as negative control. The body weight and tumor volumes calculation were recorded from the day of treatment, and the tumor volumes were calculated using the following equation: tumor volume = length × (width)^2^ × π/6. Animal experiments were reviewed and approved by the Binzhou Medical University Experimental Animal Committee.

## Results and discussion

### Structural analysis

**Scheme 1.**
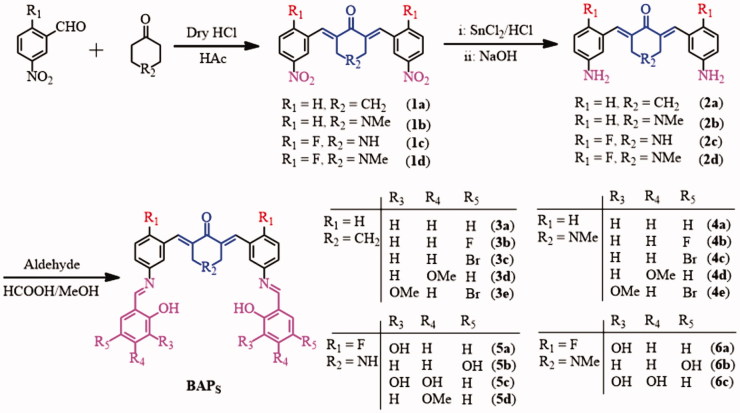
Synthetic strategy and structures of BAPs.

As shown in Scheme 1, four intermediates **2a–d** were synthesized from *m*-nitrobenzaldehyde with cyclohexanone or *N*-methyl-4-piperidinone via a Claisen-Schmidt condensation reaction, and the reduction reaction of SnCl_2_^25,26^. And then, some hydroxyl-substituted aromatic aldehydes were introduced into –NH_2_ fragment of **2a–d** to generate target compounds **3a–e, 4a–e, 5a–d**, and **6a–c** by Schiff-base condensation reaction (Scheme 1). The target compounds were purified by recrystallisation of reactive precipitation from methanol. The yields of BAPs can reach ca. 65%–78%. Their structures were confirmed by ^1^H NMR, IR and elemental analysis.

According to the ^1^H NMR spectra, the bands of amino group disappeared in about 3.9 ppm (**2a**, **2b**) and 5.15–5.32 ppm (**2c**, **2d**), while the single band in about 8.54–8.94 ppm appeared in **3a–e, 4a–e, 5a–d**, and **6a–c**, which proved the form of –CH = N bond. In 13C NMR spectra, the chemical shift of about 159 ppm should correspond to the carbon atom of –CH = N group. Hydroxyl-substituted aromatic aldehydes were condensed into Schiff-base compounds, so the chemical shift of 13.67–11.90 ppm should correspond to the *ortho*-hydroxyl proton from target compounds. In addition, the single band from –C = CH– bonds of the *α,β*-unsaturated ketone are about 8.04–7.65 ppm, which proved the existence of 1,5-diphenylpenta-1,4-dien-3-ones pharmacophore. According to the 13C NMR spectra, the band in the range of 189.79–186.39 ppm should correspond to carbonyl carbon atom, which also proved the existence of 1,5-diphenylpenta-1,4-dien-3-ones pharmacophore. In addition, cyclohexanone derivatives **3a–e** have the chemical shift of methylene that around ca. 2.98 and ca. 1.86 ppm, while 4-piperidone derivatives **4a–e,** and **6a–c** showed the chemical shifts of *N*-methyl and methylene around *δ* 2.18–3.35 ppm and *δ* 3.60–4.27 ppm. Based on the FTIR spectra, the characteristic band of 1656–1682 cm^−1^ are attributed to –C = O group of cyclohexanone or 4-piperidone. The strong bands at around 1621–1605 cm^−1^ are attributed to –C = C bonds of *α,β*-unsaturated ketone and –C = N bonds of Schiff-base groups. Additionally, element analysis results display that, the contents of C, H, N elements are basically consistent with the calculated results. It further confirms the correctness of their structures.

### Cytotoxicity analysis

The cytotoxicity activities against human carcinoma cell lines A549, SGC7901, HePG2, HeLa, K562, THP-1 and cytotoxicity toward LO2 cell lines were evaluated by MTT method. DOX and curcumin were used as positive control. The selectivity index (SI) is the quotient of the IC_50_ values towards nonmalignant LO2 cells. The results of their activity evaluation could be found in Table 1. The results suggest that IC_50_ values for about 76% of all target compounds against human carcinoma cell lines were lower than 5.0 µM. Especially for **4a–e, 5a–d**, and **6a–c**, this ratio can reach about 92%.

For cyclohexanone derivatives **2a**, the IC_50_ values against human carcinoma cell lines exceed 5.0 µM. After condensation reaction to **3a–e**, 53% of IC_50_ values were lower than 5.0 µM, especially for **3a**, **3b**, **3e**, which IC_50_ values were only 1.5 ± 0.1 µM (**3a**), 1.6 ± 0.1 µM (**3b**) against THP-1, and 1.9 ± 0.01 µM (**3e**) against HepG2. The results showed cytotoxicities of **3a–e** were better than that of **2a**. In other words, the cytotoxicities of **3a–e** were improved after Schiff-base condensation reaction. For 4-piperidone derivatives **2b** and **4a–e**, their structures changed from cyclohexanone to *N*-methyl-4-piperidinone, so the nature of *N*-substituents in the additional binding site could influence the anticancer and cytotoxic properties of the molecules^25^^,^^26^. As shown in Table 1, 2**b** displayed distinctly lower IC_50_ value than that of **2a**, and most of **4a–e** showed lower IC_50_ values (<5.0 µM) against experimental human carcinoma cell lines except **4a** against A549, SGC7901, HeLa, and **4c** against A549, K562.

By comparing the IC_50_ values of **3a–e** and **4a–e** with identical aryl substituents, 4-piperidone derivatives **4a–e** showed better cytotoxicity than cyclohexanone derivatives **3a–e**. Structure analysis showed that the biggest difference of these two series is center units, such as cyclohexanone and *N*-methyl-4-piperidinone. The results showed *N*-methyl-4-piperidinone group was more beneficial for cytotoxicity than cyclohexanone group. In addition, for the same types of derivatives, substituent effect can influence their activity. No substituent group or CH_3_O– group in R_3_ or R_4_ site of 4-piperidone derivatives should be of advantage for cytotoxicity against THP-1, such as **4a**, **4d** and **4e**.

In order to examine the substituent effect of BAPs, the strong electron-donating polyhydroxyl-substituted BAPs (**5a–d** and **6a–c**) were synthesized and evaluated (Table 1). Their IC_50_ values against experimental human carcinoma cell lines were below 5.0 µM. Especially for **5c** and **6c**, there are strong electron-donating 3,4,5-trihydroxy phenyl group showed accredited inhibitory activities against experimental human carcinoma cell lines. Compared with **3a–e** and **4a–e**, the cytotoxicities of BAPs **5a–d** and **6a–c** showed a better improvement. This may be because hydroxyl substituted BAPs are more likely to bind to bioactive groups in tumor cells. Interestingly, the more the hydroxyl groups, the better is the activity. More gratifying is 3,4,5-trihydroxyphenyl-substituted BAP **5c** which displayed the best cytotoxicity.

**Figure 2.**
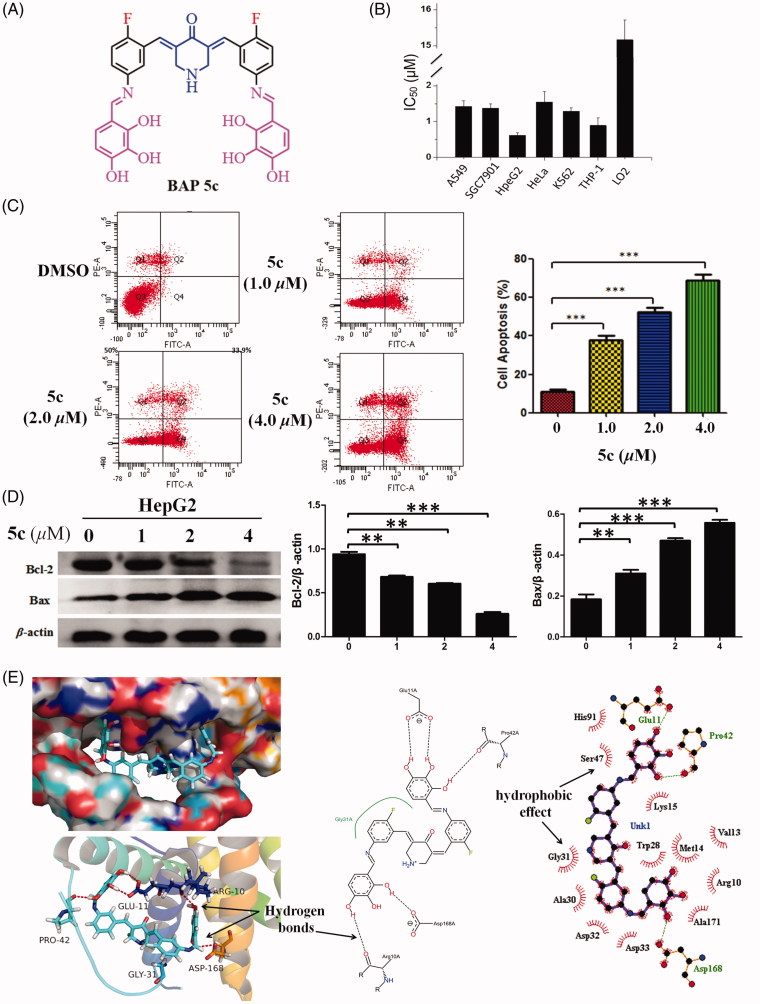
(A) The structures of BAP **5c**. (B) Cytotoxicity of BAP **5c**. (C) BAP **5c** induces HepG2 cells apoptosis. HepG2 cells were incubated with **5c** (1.0, 2.0, 4.0 µM) for 24 h and analysed for apoptosis by Annexin V-FITC staining. ***p < .001 vs the negative control. (D) The effect of **5c** on the expression level of resistance protein Bax and Bcl-2 detected by western blot. Protein was from HepG2 cell line treated for 3 h by BAP **5c** in 1.0, 2.0, 4.0 µM concentrations. The data are representative of three independent experiments. *p < .05, **p < .01, ***p < .001 vs the negative control (one-way ANOVA followed by Dunnett’s test). (E) The molecular docking modes of compound **5c** in the active site of Bcl-2 protein.

In order to study the biocompatibility of BAPs, non-malignant LO2 cell line was selected to evaluate their cytotoxicities. As shown in Table 1, all IC_50_ values toward LO2 cell line exceed 10 µM, and they were distinctly higher than that of DOX (6.1 µM) and curcumin (27.5 µM). The SI values are shown in Table 1. In consideration of cytotoxicity, the IC_50_ values of **5c** (Figure 2(A,B)) were lower than 2.0 µM against experimental human carcinoma cell lines, as well as higher SI values and lower cytotoxicity toward non-malignant LO2 cells. So BAP **5c** was selected for further biological assessment in this study.

### BAP 5c induces HepG2 cells apoptosis

To further investigate the underlying mechanism of decreased cell proliferation observed in the MTT assay, the flow cytometry were employed to determine whether HepG2 cells growth inhibition mediated by **5c** resulted from apoptosis. As shown in Figure 2(C), the results indicated that HepG2 cells showed a dose-dependent apoptosis after **5c** treatment for 24 hours, including early and late apoptosis.

### BAP 5c can regulate the expression level of Bax and Bcl-2 in HepG2

BAP **5c** was chosen to confirm the effect of the compounds synthesized on the expression level of resistance protein Bax and Bcl-2. The ratio of Bax/*β*-actin and Bcl-2/*β*-actin as the relative expression level of protein are shown in Figure 2(D). It intuitively showed the effects of pro-apoptotic from Bax and anti-apoptotic from Bcl-2 in the Bcl-2 protein family compared with the negative control, respectively. Obviously, **5c** can up-regulate the expression level of Bax, and down-regulate the expression level of Bcl-2 compared with the negative control.

### Molecular docking study of BAP 5c for Bcl-2 protein

Theoretical modeling calculations using Discovery Studio 2017R2 was performed to elucidate the mode of Bcl-2 inhibition by **5c**. There is a narrow and long groove on the surface of the anti-apoptotic Bcl-2 protein. The results showed the molecule of **5c** can be mounted on the narrow and long groove of the anti-apoptotic Bcl-2 protein through five groups of strong intermolecular O–H···O hydrogen bonds between hydroxy groups of **5c** and Glu11A, Pro42A, Arg10A, Asp168A of Bcl-2 protein (Figure 2(E)). In addition, significant hydrophobic effect between **5c** and amino acids can be found. 2-fluorinated BAP can prove the better lipophilicity than free 2-fluorinated BAPs. This could be an advantage for fluorinated BAPs as potential anticancer agents^27^^,^^28^.

### The investigation of cellular uptake *in vitro*

**Figure 3.**
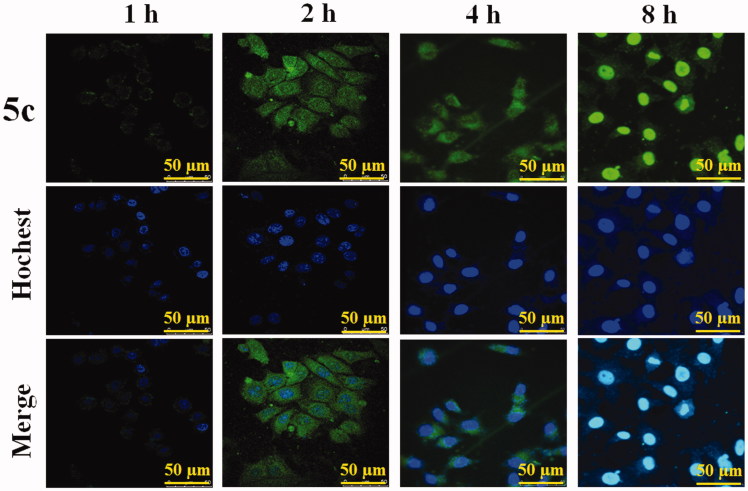
The cellular uptake of BAP **5c** in HepG2 cells at different time (1 h, 2 h, 4 h, 8 h) observed by CLSM. Scale bar: 50 µm.

Cellular uptake of BAP **5c** was detected qualitatively by confocal laser scanning microscopy (CLSM), as their fluorescence can allow the visualization inside the cells. The effect of representative **5c** on the cellular uptake of HepG2 cells is shown in Figure 3. The results showed that after treatment for 1 hour, only a little of **5c** accumulated into the nuclei; after treatment for 2 hours and 4 hours, **5c** mainly accumulated into the nuclei, while a little amount can be observed in the cytoplasma. Up to 8 hours, the distribution of **5c** was exactly opposite, and most drugs could enter into cytoplasm of HepG2 cells. Unfortunately, cell morphology disrupted following the cellular uptake of HepG2 cells, which may attribute to the cytotoxic activity of **5c**.

### BAP 5c inhibits tumor growth *in vivo*

**Figure 4.**
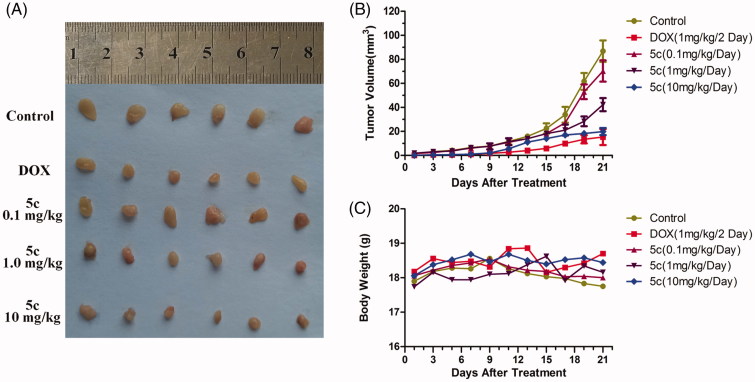
BAP **5c** inhibits HepG2 cells growth *in vivo*. (A) HepG2 cells were injected to the flanks of nude mice were allowed to develop for 3 days. Subsequently, **5c** (10 mg/kg, 1.0 mg/kg and 0.1 mg/kg) was injected daily i.p. for up to 20 days. On day 21, tumors were excised and subjected to further analyses. Representative tumors of each group were showed. (B) Tumor sizes were measured every 2 days. (C) Mice body weight was measured every 2 days.

In order to evaluate the early treatment effect of **5c** toward liver cancers, HepG2-bearing mice were used in this study. The tumor volume and body weight was measured every 2 days. After 21 days, all solid tumors were excised and photographed. The results are shown in Figure 4(A). **5c** (1.0 mg/kg) displayed light inhibitory activity While, **5c** (10 mg/kg) significantly inhibited the tumor growth, and the inhibitory effect of **5c** was similar to DOX. The tumor growth curves and the body weight curves of all groups are shown in Figure 4(B,C). In general, the tumors in control group grew continuously during the experimental period, whereas the tumor growth in the **5c** (10 mg/kg)-treated and DOX (1 mg/kg)-treated mice was suppressed significantly. However, there was no apparent change in body weight in the animals. Therefore, **5c** can be a potential anticancer agent for early treatment of liver cancers.

## Conclusions

In this study, a series of hydroxyl-substituted double Schiff-base 4-piperidone/cyclohexanone derivatives (**3a–e, 4a–e, 5a–d**, and **6a–c**) were synthesized and characterised. Their biological activities as potential anticancer agents were evaluated primarily. Hydroxyl-substituted 4-piperidinone derivatives showed better anticancer activity than cyclohexanone derivatives. Especially for **5c**, the IC_50_ values are lower than 2.0 µM against experimental human carcinoma cell lines, as well as lower cytotoxicity toward non-malignant LO2 cells. The western blot results proved **5c** can effectively promote cell apoptosis through up-regulating Bax protein and down-regulating Bcl-2 protein expression. Molecular docking modes showed **5c** could reasonably bind to the active site of Bcl-2 protein through strong intermolecular hydrogen bonds and significant hydrophobic effect. *In vivo*, BAP **5c** can effectively suppress the growth of HepG2 xenografts without apparent body weight changes. These data indicate that BAP **5c** can be a potential anticancer agent for early treatment of liver cancers.

## Supplementary Material

Supporting_information.doc
