# Supplementary material for: Hydroxyl-substituted double Schiff-base condensed 4-piperidone/cyclohexanones as potential anticancer agents with biological evaluation
Source: J Enzyme Inhib Med Chem. 2019 Jan 2;34(1):264–71. doi: 10.1080/14756366.2018.1501042 (PMC6327999; doi:10.1080/14756366.2018.1501042)
Supplement: Supporting_information.doc [file IENZ_A_1501042_SM7984.doc]

**Supporting Information**

**Hydroxyl-substituted double Schiff-base condensed 4-piperidone/cyclohexanone****s as potential anticancer agents with biological evaluation**

Lianshuang Zhang^1^, Qin Chen^2^, Guige Hou^1^*, Wei Zhao^1^, Yun Hou^1^*

*^1^ Binzhou Medical University, Yantai, Shandong Province, 264003, P. R. China and ^2^ Department of Pharmacy, Taihe County People’s Hospital, Taihe, Anhui Province, 236600, P. R. China*

*Tel: +86-535-6913406; fax: +86-535-6913718;*

******E-mail address: guigehou@163.com (G.-G. Hou), houyun820424@163.com (Y. Hou)*

**Structural characterization**

**2c**: Light yellow powder; Yield: 51%; mp: 107-109 ℃. IR (cm^-1^): 3341(br), 1668(s), 1610(s), 1590(m), 1491(s), 1445(m), 1350(s), 1310(m), 1261(s), 1233(m), 1216(s), 1195(m), 1158(m), 1003(s), 932(s), 866(s), 816(s), 643(s), 564(s), 507(s). ^1^H NMR (400 MHz, DMSO): *δ* 7.55 (s, 2H), 6.94 (d, *J* = 8.8 Hz, 2H), 6.62 (s, 4H), 5.15 (s, 4H), 3.89 (s, 4H). ^13^C NMR (150 MHz, DMSO) δ 187.70 (s), 153.28 (d, *J* = 236.8 Hz), 145.54 (s), 137.22 (s), 126.97 (d, *J* = 4.1 Hz), 122.73 (d, *J* = 13.3 Hz), 116.55 (d, *J* = 7.5 Hz), 116.12 (d, *J* = 22.9 Hz), 115.17 (d, *J* = 20.5 Hz), 48.16 (s). Elemental analysis (%) caled. for C_19_H_17_F_2_N_3_O (341.35): C 66.85, H 5.02, N 12.31. Found: C 66.89, H 5.06, N 12.27.

**2d**: Light yellow powder; Yield: 46%; mp: 124-126 ℃. IR (cm^-1^): 3343(s), 2941(s), 2787(s), 1672(s), 1618(s), 1592(m), 1493(s), 1448(m), 1306(m), 1269(s), 1251(s), 1215(s), 1180(s), 1160(s), 1128(s), 1100(s), 1003(s), 925(s), 867(s), 813(s), 694(s). ^1^H NMR (400 MHz, DMSO): *δ* 7.74 (s, 2H), 7.46 (d, *J* = 7.8 Hz, 2H), 6.97 (d, *J* = 7.8 Hz, 2H), 6.46 (s, 2H), 5.32 (s, 4H), 3.86 (s, 4H), 3.35 (s, 3H). ^13^C NMR (150 MHz, DMSO) δ 186.63 (s), 150.40 (d, *J* = 233.9 Hz), 142.50 (s), 132.90 (s), 131.62 (d, *J* = 30.0 Hz), 123.96 (d, *J* = 8.2 Hz), 117.06 (d, *J* = 16.7 Hz), 115.94 (d, *J* = 21.5 Hz), 112.94 (d, *J* = 20.7 Hz), 56.40 (s), 45.97 (s). Elemental analysis (%) caled. for C_20_H_19_F_2_N_3_O (355.38): C 67.59, H 5.39, N 11.82. Found: C 67.51, H 5.37, N 11.79.

**3a**: Light yellow powder; Yield: 72%; mp: 133~135℃. IR(cm^-1^): 1667(s), 1619(s), 1596(m), 1568(s), 1282(s), 1169(s), 1149(s), 1031(s), 982(s), 820(m), 804(s), 794(m), 747(s), 734(m), 695(m). ^1^H NMR (400 MHz, CDCl_3_): δ 13.13 (s, 2H), 8.66 (s, 2H), 7.84 (s, 2H), 7.50-7.20(m, 12H), 7.07 (d, *J* = 8.0 Hz, 2H), 6.98 (t, *J* = 8.0 Hz, 2H), 2.99 (t, *J* = 4.0 Hz, 4H), 1.85 (m, 2H). ^13^C NMR (100 MHz, DMSO): δ 188.91, 164.19, 160.24, 148.45, 136.95, 136.59, 135.32, 133.47, 132.65, 129.65, 128.57, 123.01, 121.96, 119.29, 119.20, 116.61, 27.85, 22.33. Elemental analysis (%) calcd. for C_34_H_28_N_2_O_3_ (512.59): C 79.67, H 5.51, N 5.46; Found: C 79.78, H 5.45, N 5.42.

**3b**: Deep yellow powder; Yield: 67%; mp: 152~154℃. IR(cm^-1^): 1656(m), 1621(s), 1568(s), 1489(s), 1356(m), 1278(s), 1214(m), 1173(m), 1140(s), 866(s), 817(m), 786(s), 690(s). ^1^H NMR (400 MHz, CDCl_3_): δ 12.87 (s, 2H), 8.60 (s, 2H), 7.84 (s, 2H), 7.50 (t, *J* = 7.7 Hz 2H), 7.43 (d, *J* = 7.6 Hz 2H), 7.37 (s, 2H), 7.27 (s, 2H), 7.14 (d, *J* = 8.0 Hz 4H), 7.02 (m, 2H), 2.98 (t, *J* = 5.1 Hz 4H), 1.85 (m, 2H). ^13^C NMR (100 MHz, DMSO): δ 188.88, 162.67, 156.41, 156.10, 153.77, 148.48, 136.75, 135.26, 129.68, 128.80, 123.02, 121.90, 120.40, 119.62, 118.04, 116.98, 27.85, 22.32. Elemental analysis (%) calcd. for C_34_H_26_F_2_N_2_O_3_ (528.57): C 74.44, H 4.78, N 5.11; Found: C 74.38, H 4.73, N 5.17.

**3c**: Orange powder; Yield: 69%; mp: 231~233℃; IR(cm^-1^): 3229(br), 1672(s), 1619(m), 1564(m), 1466(s), 1873(m), 1275(s), 1208(m), 1168(s), 1154(s), 1116(m), 1071(m), 892(s), 884(s), 828(s), 766(s), 709(s), 694(s). ^1^H NMR (400 MHz, CDCl_3_): δ 13.13 (s, 2H), 8.59 (s, 2H), 7.84 (s, 2H), 7.58-7.54 (m, 2H), 7.50 (d, J = 8.3 Hz 4H), 7.43 (d, J = 7.5 Hz 2H), 7.36 (s, 2H), 7.28 (s, 2H), 6.98 (d, *J* = 8.8 Hz 2H ), 2.98 (t, *J* = 5.1 Hz 4H), 1.86 (m, 2H). ^13^C NMR (100 MHz, DMSO): δ 188.90, 162.43, 155.25, 154.68, 152.41, 147.21, 136.82, 135.14, 128.89, 123.43, 121.27, 120.40, 119.36, 118.10, 117.21, 115.89, 27.85, 22.33. Elemental analysis (%) calcd. for C_34_H_26_Br_2_N_2_O_3_ (670.38): C 60.91, H 3.91, N 4.18; Found: C 60.94, H 3.87, N 4.17.

**3d**: Yellow powder; Yield: 73%; mp: 160~162℃; IR(cm^-1^): 1662(s), 1614(s), 1595(m), 1565(s), 1513(s), 1444(s), 1399(m), 1343(m), 1286(s), 1210(s), 1168(s), 1118(s), 1028(m), 967(m), 939(s), 884(s), 829(s), 797(s), 689(s). ^1^H NMR (400 MHz, CDCl_3_): δ 13.56 (s, 2H), 8.57 (s, 2H), 7.82 (s, 2H), 7.47 (t, *J* = 7.6 Hz, 2H), 7.40-7.30 (m, 6H), 7.26 (s, 2H), 6.61 (s, 2H), 6.55 (d, *J* = 8.5 Hz, 2H), 3.88 (s, 6H), 2.98 (t, *J* = 4.0 Hz, 4H), 1.89 (m, 2H). ^13^C NMR (100 MHz, DMSO): δ 188.92, 163.77, 163.30, 163.07, 148.23, 136.88, 136.57, 135.39, 134.25, 129.62, 128.10, 122.79, 121.77, 112.97, 106.91, 100.79, 55.50, 27.86, 22.34. Elemental analysis (%) calcd. for C_36_H_32_N_2_O_5_ (572.64): C 75.51, H 5.63, N 4.89; Found: C 75.58, H 5.71, N 4.93.

**3e**: Orange powder; Yield: 70%; mp: 136~138℃; IR(cm^-1^): 1664(m), 1615(s), 1567(m), 1469(s), 1440(s), 1390(s), 1361(s), 1325(s), 1227(s), 1251(s), 1209(m), 1171(s), 1147(m), 976(s), 866(m), 835(m), 789(m), 685(s). ^1^H NMR (400 MHz, CDCl_3_): δ 13.67 (s, 2H), 8.59 (s, 2H), 7.83 (s, 2H), 7.50 (t, *J* = 7.7 Hz, 2H), 7.43 (d, *J* = 7.6 Hz, 2H), 7.37 (s, 2H), 7.28 (s, 2H), 7.19 (s, 2H), 7.09 (s, 2H), 3.95 (s, 6H), 2.97 (t, *J* = 4.0 Hz, 4H), 1.88 (m, 2H). ^13^C NMR (100 MHz, DMSO): δ 189.79, 162.83, 149.93, 149.16, 147.97, 137.03, 136.62, 135.23, 129.72, 128.93, 125.15, 122.93, 121.10, 120.32, 117.77, 109.36, 56.28, 27.85, 22.38. Elemental analysis (%) calcd. for C_36_H_30_Br_2_N_2_O_5_ (730.44): C 59.20, H 4.14, N 3.84; Found: C 59.23, H 4.17, N 3.79.

**4a**: Light yellow powder; Yield: 69%; mp: 114~116℃; IR(cm^-1^): 1675(s), 1615(m), 1563(m), 1278(m), 1182(s), 1152(s), 1102(s), 1058(s), 974(s), 922(s), 881(s), 800(s), 743(m), 691(m). ^1^H NMR (400 MHz, CDCl_3_): δ 12.95 (s, 2H), 8.45 (s, 2H), 7.65 (s, 2H), 7.40-7.00 (m, 12H), 6.95-6.70 (m, 4H), 3.70-3.50 (m, 4H), 2.30 (s, 3H). ^13^C NMR (100 MHz, CDCl_3_): δ 186.62, 163.47, 161.08, 148.94, 136.49, 135.80, 133.73, 133.48, 132.46, 129.63, 128.58, 122.99, 121.58, 119.22, 119.05, 117.29, 56.98, 45.89. Elemental analysis (%) calcd. for C_34_H_29_N_3_O_3_ (527.61): C 77.40, H 5.54, N 7.96; Found: C 77.43, H 5.53, N 7.94.

**4b**: Yellow powder; Yield: 67%; mp: 62~64℃; IR(cm^-1^): 2937(br), 1672(s), 1617(m), 1568(s), 1488(m), 1354(s), 1327(s), 1277(s), 1244(m), 1213(m), 1182(s), 1141(s), 1102(s), 1056(m), 1033(s), 1006(s), 908(s), 867(s), 822(s), 797(s), 784(s), 687(m). ^1^H NMR (400 MHz, CDCl_3_): δ 12.79 (s, 2H), 8.60 (s, 2H), 7.98 (s, 2H), 7.53 (t, *J* = 7.78 Hz, 2H), 7.33 (d, *J* =7.8 Hz, 4H), 7.27 (s, 2H), 7.15 (d, *J* = 7.9 Hz, 4H), 7.02(q, *J* = 4.0 Hz, 2H), 4.02 (s, 4H), 2.59 (s, 3H). ^13^C NMR (100 MHz, DMSO): δ 186.43, 162.70, 156.26, 153.80, 148.71, 135.91, 134.29, 129.86, 128.64, 123.29, 122.21, 120.53, 120.30, 119.68, 118.06, 116.85, 56.23, 45.20. Elemental analysis (%) calcd. for C_34_H_27_F_2_N_3_O_3_ (563.59): C 72.46, H 4.83, N 7.46; Found: C 72.45, H 4.85, N 7.43.

**4c**: Yellow powder; Yield: 78%; mp: 143~145℃; IR(cm^-1^): 1672(s), 1614(s), 1593(s), 1561(s), 1476(m), 1352(m), 1277(s), 1225(m), 1182(s), 1103(m), 1107(m), 936(s), 907(m), 810(s), 794(s), 713(s), 683(s). ^1^H NMR (400 MHz, CDCl_3_): δ 13.02 (s, 2H), 8.59 (s, 2H), 8.04 (s, 2H), 7.59-7.46 (m, 6H), 7.32 (t, *J* = 4.0 Hz, 4H), 7.27 (s, 2H), 6.97 (d, *J* = 8.8 Hz, 2H), 4.13 (s, 4H), 2.62 (s, 3H). ^13^C NMR (100 MHz, DMSO): δ 186.39, 162.16, 156.02, 153.46, 148.36, 135.87, 134.16, 129.66, 128.43, 123.21, 122.15, 120.50, 120.25, 119.53, 118.00, 116.38, 56.21, 45.19. Elemental analysis (%) calcd. for C_34_H_27_Br_2_N_3_O_3_ (685.40): C 59.58, H 3.97, N 6.13; Found: C 59.61, H 3.93, N 6.11.

**4d**: Yellow powder; Yield: 73%; mp: 120~122℃; IR(cm^-1^): 1672(m), 1614(s), 1590(m), 1563(s), 1512(s), 1399(s), 1434(s), 1287(m), 1210(s), 1181(m), 1163(s), 1135(m), 1115(m), 1055(s), 1031(s), 966(s), 915(s), 834(m), 791(m), 686(m). ^1^H NMR (400 MHz, CDCl_3_): δ 13.57 (s, 2H), 8.56 (s, 2H), 7.89 (s, 2H), 7.50 (t, *J* = 7.6 Hz, 2H), 7.36-7.20 (m, 8H), 6.54 (d, *J* = 7.0 Hz, 4H), 3.87 (m, 10H), 2.18 (s, 3H). ^13^C NMR (100 MHz, DMSO): δ 186.42, 163.83, 163.38, 163.06, 148.44, 135.88, 134.40, 134.23, 134.19, 129.79, 127.95, 123.07, 122.10, 113.01, 106.93, 100.84, 56.24, 55.51, 45.20. Elemental analysis (%) calcd. for C_36_H_33_N_3_O_5_ (587.66): C 73.58, H 5.66, N 7.15; Found: C 73.67, H 5.72, N 7.21.

**4e**: Orange powder; Yield: 65%; mp: 140~142℃; IR(cm^-1^): 1672(m), 1614(m), 1590(s), 1567(m), 1469(s), 1394(s), 1330(s), 1276(s), 1253(s), 1182(s), 1104(s), 973(m), 908(s), 865(m), 832(m), 791(s), 751(s), 684(m). ^1^H NMR (400 MHz, CDCl_3_): δ 13.60 (s, 2H), 8.58 (s, 2H), 7.87 (s, 2H), 7.51 (t, *J* = 7.7 Hz, 2H), 7.37-7.27 (m, 6H), 7.19 (s, 2H), 7.09 (s, 2H), 3.95 (s, 6H), 3.86 (s, 4H), 2.51 (s, 3H). ^13^C NMR (100 MHz, DMSO): δ 189.88, 162.84, 149.95, 149.21, 148.19, 135.91, 134.32, 129.89, 128.76, 125.11, 123.27, 122.23, 120.40, 117.88, 109.38, 56.32, 56.17, 45.15. Elemental analysis (%) calcd. for C_36_H_31_Br_2_N_3_O_5_ (745.45): C 58.00, H 4.19, N 5.64; Found: C 57.95, H 4.22, N 5.65.

**5a**: Orange powder; Yield: 72%; mp: 178~180 ℃. IR (cm^-1^): 3070(br), 2748(s), 1662(m), 1618(m), 1488(m), 1463(m), 1365(s), 1260(s), 1202(s), 1151(s), 1106(s), 1077(s), 1006(s), 964(s), 936(s), 852(s), 824(s), 785(m), 735(s), 588(s). ^1^H NMR (400 MHz, DMSO) : *δ* 12.79 (s, 2H), 9.30 (s, 2H), 8.94 (s, 2H), 7.79 (s, 2H), 7.66-7.36 (m, 6H), 7.11 (d, *J* = 7.4 Hz, 2H), 6.98 (d, *J* = 7.5 Hz, 2H), 6.81 (t, *J* = 7.8 Hz, 2H), 4.24 (s, 4H). ^13^C NMR (150 MHz, DMSO) δ 187.51 (s), 164.99 (s), 159.42 (d, *J* = 248.9 Hz), 149.56 (s), 146.09 (s), 144.94 (s), 138.57 (s), 126.22 (d, *J* = 7.5 Hz), 124.53 (d, *J* = 8.6 Hz), 123.94 (s), 123.80 (d, *J* = 14.7 Hz), 123.28 (s), 119.73 (d, *J* = 40.3 Hz), 119.33 (s), 117.13 (d, *J* = 23.4 Hz), 115.12 (s), 47.83 (s). Elemental analysis (%) calcd. for C_33_H_25_F_2_N_3_O_5_ (581.56): C 68.15, H 4.33, N 7.23; Found: C 68.13, H 4.31, N 7.27.

**5b**: Orange powder; Yield: 70%; mp: 195~197 ℃. IR (cm^-1^): 3174(s), 2780(s), 1681(s), 1618(m), 1585(s), 1487(s), 1462(s),1210(s), 1165(m), 1146(m), 1049(s), 1009(s), 986(m), 954(s), 884(m), 850(s), 826(s), 790(m), 743(s), 711(m), 612(s). ^1^H NMR (400 MHz, DMSO) : *δ* 11.90 (s, 2H), 9.19 (s, 2H), 8.87 (s, 2H), 7.86 (s, 2H), 7.58 (s, 2H), 7.54-7.41 (m, 4H), 7.07 (d, *J* = 2.0 Hz, 2H), 6.89 (dd, *J* = 8.7, 2.3 Hz, 2H), 6.82 (d, *J* = 8.8 Hz, 2H), 4.38 (s, 4H). ^13^C NMR (150 MHz, DMSO) δ 187.43 (s), 164.18 (s), 163.67 (s), 159.35 (d, *J* = 248.7 Hz), 153.44 (s), 150.14 (s), 145.53 (s), 138.38 (s), 126.43 (d, *J* = 8.2 Hz), 124.56 (d, *J* = 8.7 Hz), 123.96 (s), 123.72 (d, *J* = 14.8 Hz), 121.78 (s), 119.76 (s), 117.51 (d, *J* = 58.8 Hz), 117.10 (d, *J* = 23.3 Hz), 47.74 (s). Elemental analysis (%) calcd. for C_33_H_25_F_2_N_3_O_5_ (581.56): C 68.15, H 4.33, N 7.23; Found: C 68.13, H 4.38, N 7.19.

**5c**: Reddish brown powder; Yield: 78%; mp: 226~228 ℃. IR (cm^-1^): 3063(s), 2936(s), 2642(s), 1665(s), 1616(s), 1581(s), 1492(s), 1448(m), 1300(s), 1265(s), 1192(s), 1081(m), 994(m), 928(s), 886(s), 814(m), 800(m), 713(s), 668(s), 566(m). ^1^H NMR (400 MHz, DMSO) : *δ* 13.23 (s, 2H), 9.84 (s, 2H), 8.79 (s, 2H), 8.52 (s, 2H), 7.76 (s, 2H), 7.51 (s, 2H), 7.42 (t, *J* = 8.7 Hz, 4H), 6.97 (d, *J* = 8.3 Hz, 2H), 6.46 (d, *J* = 8.4 Hz, 2H), 4.16 (s, 4H). ^13^C NMR (150 MHz, DMSO) δ 187.37 (s), 164.43 (s), 163.89 (s), 159.02 (d, *J* = 248.4 Hz), 151.37 (s), 151.03 (s), 145.03 (d, *J* = 2.2 Hz), 138.25 (s), 132.86 (s), 124.70 (d, *J* = 7.9 Hz), 124.26 (d, *J* = 8.6 Hz), 123.71 (d, *J* = 14.8 Hz), 123.58 (s), 117.09 (d, *J* = 23.3 Hz), 112.81 (s), 108.33 (s), 47.74 (s). Elemental analysis (%) calcd. for C_33_H_25_F_2_N_3_O_7_ (613.56): C 64.60, H 4.11, N 6.85; Found: C 64.63, H 4.14, N 6.81.

**5d**: Yellow powder; Yield: 72%; mp: 163~165 ℃. IR (cm^-1^): 2620(br), 1682(s), 1613(s), 1512(m), 1488(m), 1460(s), 1362(s), 1343(s), 1295(m), 1257(m), 1206(s), 1166(m), 1137(m), 937(s), 890(s), 850(s), 816(m), 801(m), 770(s), 734(s), 564(s). ^1^H NMR (400 MHz, DMSO): *δ* 13.27 (s, 2H), 8.90 (s, 2H), 7.85 (s, 2H), 7.55 (d, *J* = 8.6 Hz, 4H), 7.48 (dd, *J* = 10.5, 5.5 Hz, 4H), 6.59 (d, *J* = 8.6 Hz, 2H), 6.51 (d, *J* = 1.7 Hz, 2H), 4.38 (s, 4H), 3.81 (s, 6H). ^13^C NMR (150 MHz, DMSO) δ 187.07 (s), 164.21 (s), 163.75 (s), 163.24 (s), 159.15 (d, *J* = 248.6 Hz), 145.01 (s), 137.85 (s), 134.66 (s), 126.75 (d, *J* = 8.1 Hz), 124.34 (d, *J* = 8.8 Hz), 123.73 (s), 123.64 (d, *J* = 14.9 Hz), 117.07 (d, *J* = 23.4 Hz), 113.43 (s), 107.36 (s), 101.25 (d, *J* = 8.2 Hz), 55.95 (s), 47.57 (s). Elemental analysis (%) calcd. for C_35_H_29_F_2_N_3_O_5_ (609.61): C 68.96, H 4.79, N 6.89; Found: C 68.99, H 4.83, N 6.85.

**6a**: Yellow powder; Yield: 68%; mp: 159~161 ℃. IR (cm^-1^): 3072(s), 1677(m), 1619(s), 1581(m), 1485(m), 1462(m), 1270(s), 1205(m), 1182(s), 973(s), 933(s), 884(s), 852(s), 823(s), 794(s), 783(s). ^1^H NMR (400 MHz, DMSO): *δ* 12.81 (s, 2H), 9.28 (s, 2H), 8.93 (s, 2H), 7.75 (s, 2H), 7.56 (s, 2H), 7.46 (dd, *J* = 19.0, 8.1 Hz, 4H), 7.11 (d, *J* = 7.4 Hz, 2H), 6.97 (d, *J* = 7.5 Hz, 2H), 6.81 (t, *J* = 7.7 Hz, 2H), 3.88 (s, 4H), 3.35 (s, 3H). ^13^C NMR (150 MHz, DMSO) δ 186.66 (s), 163.59 (s), 162.98 (s), 157.51 (d, *J* = 245.9 Hz), 149.56 (s), 146.07 (s), 140.85 (s), 134.37 (s), 130.72 (d, *J* = 8.5 Hz), 124.43 (d, *J* = 7.9 Hz), 123.97 (s), 123.55 (d, *J* = 15.9 Hz), 123.13 (s), 119.94 (d, *J* = 22.9 Hz), 119.22 (d, *J* = 23.2 Hz), 112.57 (s), 56.48 (s), 45.59 (s). Elemental analysis (%) calcd. for C_34_H_27_F_2_N_3_O_5_ (595.59): C 68.56, H 4.57, N 7.06; Found: C 68.62, H 4.50, N 7.12.

**6b**: Yellow powder; Yield: 76%; mp: 173~175 ℃. IR (cm^-1^): 3092(s), 1681(s), 1605(s), 1578(m), 1476(m), 1459(m), 1411(s), 1355(s), 1270(s), 1198(m), 1173(s), 961(s), 921(s), 874(s), 803(s), 787(s), 735(s), 683(s). ^1^H NMR (400 MHz, DMSO): *δ* 11.92 (s, 2H), 9.19 (s, 2H), 8.87 (s, 2H), 7.84 (s, 2H), 7.52 (d, *J* = 37.9 Hz, 6H), 7.03 (d, *J* = 33.7 Hz, 2H), 6.85 (d, *J* = 22.9 Hz, 4H), 4.27 (s, 4H), 2.73 (s, 3H). ^13^C NMR (150 MHz, DMSO) δ 186.65 (s), 163.53 (s), 162.31 (s), 157.43 (d, *J* = 253.4 Hz), 153.39 (s), 150.09 (s), 141.45 (s), 138.38 (s), 124.38 (d, *J* = 7.6 Hz), 124.22 (d, *J* = 8.2 Hz), 123.37 (d, *J* = 16.6 Hz), 121.26 (s), 119.85 (s), 117.59 (d, *J* = 38.8 Hz), 117.32 (d, *J* = 22.6 Hz), 112.52 (s), 56.48 (s), 45.56 (s). Elemental analysis (%) calcd. for C_34_H_27_F_2_N_3_O_5_ (595.59): C 68.56, H 4.57, N 7.06; Found: C 68.53, H 4.62, N 7.03.

**6c**: Yellow powder; Yield: 78%; mp: 205~207 ℃. IR (cm^-1^): 3075(br), 1680(s), 1620(s), 1591(s), 1558(s), 1494(m), 1457(m), 1268(s), 1205(m), 1171(m), 1081(m), 990(m), 884(s), 811(m), 773(s), 696(s), 631(s), 594(s). ^1^H NMR (400 MHz, DMSO): *δ* 13.24 (s, 2H), 9.82 (s, 2H), 8.79 (s, 2H), 8.54 (s, 2H), 7.74 (s, 2H), 7.46 (d, *J* = 35.8 Hz, 6H), 6.97 (d, *J* = 7.9 Hz, 2H), 6.46 (d, *J* = 8.0 Hz, 2H), 3.86 (s, 4H), 3.35 (s, 3H). ^13^C NMR (150 MHz, DMSO) δ 186.65 (s), 163.68 (s), 162.51 (s), 157.01 (d, *J* = 237.8 Hz), 151.49 (s), 150.58 (s), 140.95 (d, *J* = 5.7 Hz), 134.25 (s), 132.89 (d, *J* = 6.4 Hz), 130.81 (s), 124.36 (d, *J* = 4.4 Hz), 123.55 (d, *J* = 15.9 Hz), 123.18 (d, *J* = 24.4 Hz), 112.88 (s), 112.57 (s), 108.18 (s), 56.45 (s), 45.61 (s). Elemental analysis (%) calcd. for C_34_H_27_F_2_N_3_O_7_ (627.59): C 65.07, H 4.34, N 6.70; Found: C 65.09, H 4.31, N 6.72.
